# Rapid growth in disposable e‐cigarette vaping among young adults in Great Britain from 2021 to 2022: a repeat cross‐sectional survey

**DOI:** 10.1111/add.16044

**Published:** 2022-09-11

**Authors:** Harry Tattan‐Birch, Sarah E. Jackson, Loren Kock, Martin Dockrell, Jamie Brown

**Affiliations:** ^1^ Department of Behavioural Science and Health University College London London UK; ^2^ SPECTRUM Consortium London UK; ^3^ Addictions and Inclusion, Office for Health Improvement and Disparities London UK

**Keywords:** Disposable e‐cigarettes, electronic nicotine delivery systems, Elf Bar, ENDS, England, Puff Bar, Scotland, vaping, Wales, young adults

## Abstract

**Aims:**

To estimate recent trends in the prevalence of disposable e‐cigarette vaping in Great Britain, overall and across ages, and to measure these trends in the context of changes in smoking and vaping prevalence.

**Design:**

The Smoking Toolkit Study, a monthly representative cross‐sectional survey.

**Setting:**

Great Britain.

**Participants:**

A total of 36 876 adults (≥ 18 years) completed telephone interviews between January 2021 and April 2022.

**Measurements:**

Current e‐cigarette vapers were asked which type of device they mainly use. We estimated age‐specific monthly time trends in the prevalence of current disposable e‐cigarette use among vapers and inhaled nicotine use (vaping/smoking), smoking and vaping among adults.

**Findings:**

From January 2021 to April 2022, there was an 18‐fold increase in the percentage of vapers who used disposables, rising from 1.2 to 22.2% [prevalence ratio (PR) = 18.0; 95% compatibility interval (CI) = 9.18–49.0]. Growth in disposable e‐cigarette vaping was most pronounced in younger adults (interaction *P*‐value = 0.013): for example, the percentage of 18‐year‐old vapers using disposables rose from 0.4 to 54.8% (PR = 129; 95% CI = 28.5–4520), while it rose from 2.1 to 10.0% (PR = 4.73; 95% CI = 2.06–23.6) among 45‐year‐old vapers. However, the overall percentage of people currently using any inhaled nicotine remained stable over time both among all adults (20.0 versus 21.2%; PR = 1.06; 95% CI = 0.92–1.22) and among 18‐year‐olds (30.2 versus 29.7%; PR = 0.99; 95% CI = 0.80–1.22). In 18‐year‐olds, vaping prevalence grew (11.3 versus 17.7%; PR = 1.57; 95% CI = 1.12–2.29), and there was imprecise evidence for a decline in smoking (24.5 versus 19.5%; PR = 0.80; 95% CI = 0.63–1.04). In 45‐year‐olds, there was relatively little change in vaping (PR = 1.08; 95% CI = 0.88–1.33) or smoking prevalence (PR = 1.01; 95% CI = 0.88–1.16).

**Conclusions:**

Use of disposable e‐cigarettes in Great Britain grew rapidly between 2021 and 2022, especially among younger adults, but the overall prevalence of inhaled nicotine use was stable over time. Most young adult vapers in Great Britain now use disposable products.

## INTRODUCTION

Early electronic cigarettes (‘e‐cigarettes’) were disposable products that were poor at delivering nicotine. Over time, new e‐cigarette types were developed to deliver nicotine contained in e‐liquid more effectively through rechargeable devices with refillable tanks or replaceable pods (e.g. Juul) [1]. These devices came to dominate the global e‐cigarette market and, by 2019, fewer than one in 10 vapers used disposables in England or the United States [1, 2, 3]. Recently, a new form of disposable e‐cigarette has started being sold under brand names such as ‘Puff bar’, ‘Elf bar’ or ‘Geek bar’ [4]. Unlike earlier disposables, these products deliver nicotine effectively using a similar technology to pod devices, including high‐strength (20 mg/ml in UK/EU) nicotine salts e‐liquid [5]. They retail for approximately £5–7 (US$7–9) in the United Kingdom—about half the price of a pack of 20 cigarettes. US data show that, in 2021, disposables surpassed pods as the most commonly used type of e‐cigarette among adolescents [2]. Little is known about the popularity of disposables in other countries and older age groups. It is also unclear whether these products attract people who would otherwise smoke cigarettes, vape other types of e‐cigarettes or who would remain abstinent from nicotine entirely. This study aims to estimate recent trends in the prevalence of disposable e‐cigarette vaping in Great Britain, overall and across ages, and to explore these trends in the context of other changes in smoking and vaping prevalence.

## METHODS

### Design

The Smoking Toolkit Study (STS) is a monthly cross‐sectional survey that recruits a nationally representative sample of adults (≥ 18 years) in Great Britain. It uses a hybrid of population and quota sampling. Great Britain is divided into areas of approximately 300 households, which are stratified by region and demographic profile before being selected at random to be included on the interview list. In selected areas, interviews are performed with one individual per household until age, employment status and gender quotas are met. Raking is used to construct survey weights, adjusting data so that the demographic profile of the weighted sample matches that of Great Britain. This demographic profile is ascertained monthly using data from three sources: the 2011 UK Census, the Office for National Statistics mid‐year estimates and the annual National Readership Survey. Methods are described in detail elsewhere [6].

### Participants

Participants (*n* = 36 876) completed telephone interviews between January 2021 and April 2022, inclusive. University College London Ethics Committee provided approval for the study (0498/001), and participants gave oral informed consent. All methods were carried out in accordance with relevant regulations.

### Measures

All measures used were routinely collected in the STS. Smoking status was ascertained by asking participants which of the following applies to them: (i) ‘I smoke cigarettes (including hand‐rolled) every day’, (ii) ‘I smoke cigarettes (including hand‐rolled), but not every day’, (iii) ‘I do not smoke cigarettes at all, but I do smoke tobacco of some kind (e.g. pipe, cigar or shisha)’, (iv) ‘I have stopped smoking completely in the last year’, (v) ‘I stopped smoking completely more than a year ago’ and (vi) ‘I have never been a smoker (i.e. smoked for a year or more)’. Participants were told that this question referred to cigarettes and other kinds of tobacco, not e‐cigarettes or heat‐not‐burn products. Participants selecting (i) to (iii) were classified current smokers, (iv) and (v) former smokers and (vi) never smokers.

Vaping status was assessed by asking participants whether they were currently using e‐cigarettes to cut down on the amount they smoke, in situations when they are not allowed to smoke, to help them stop smoking or for any other reason. Those who responded positively to any of these questions were considered current vapers.

Current vapers were asked which type of device they mainly use. Those who responded, ‘a disposable e‐cigarette or vaping device (non‐rechargeable)’ were considered disposable e‐cigarette vapers. They could only choose one device type (the one they ‘mainly’ use).

Participants were asked to provide their exact age in years. Those who refused to give their exact age were asked to select their age group from a list. For participants who only responded to the latter question (2% of respondents), exact age was imputed as the mean age within the age group they selected. Participants were also asked for their gender.

### Analysis

Weighted logistic regression was used to estimate monthly time trends in the proportion of (i) adults and (ii) current vapers who use disposable e‐cigarettes, overall and for specific ages (using survey weights described earlier). For the overall analysis, models only included predictors for time. For the age‐specific analysis, models included time, age and their interaction as predictors—thus allowing for time trends to differ across ages. Both age and time were modelled continuously using restricted cubic splines with three knots (placed at earliest, middle and latest month for time and 5, 50 and 95% quantiles for age among vapers). This allowed the relationship of prevalence with age and time to be flexible and non‐linear, while avoiding categorization [7]. Age was modelled continuously, so we displayed estimates for four specific ages (18‐, 25‐, 35‐ and 45‐year‐olds) to illustrate how trends differed across ages. Note that the model used to derive these estimates included data from participants of all ages, not only those who were aged exactly 18, 25, 35 or 45 years.

Prevalence ratios (PR) for the change in prevalence across the whole time‐series (April 2022 versus January 2021) were presented, alongside 95% compatibility intervals (95% CIs) calculated using bootstrapping [8, 9, 10, 11]. We ran analogous analyses to estimate time trends in the proportion of adults who currently (i) vape, (ii) smoke or (iii) use any form of inhaled nicotine—whether smoked or vaped. Note that prevalence of disposable e‐cigarette use was very low in older age groups, which meant that we were unable to estimate time trends in these groups. Finally, we reported the percentage of disposable e‐cigarette vapers who reported being current, former or never smokers. Participants with missing data for their smoking or vaping status (< 1%) were excluded from analyses that required this information. R version 4.1.0 was used for analyses (code: https://osf.io/km3x6/).

## RESULTS

Of the 36 876 eligible adults interviewed, 51.1% were women and the average age was 51.5 years [standard deviation (SD) = 18.6]. From January 2021 to April 2022, there was an 18‐fold increase in the percentage of vapers who used disposables, rising from 1.2 to 22.2% [prevalence ratio (PR) = 18.0; 95% compatibility interval (CI) = 9.18–49.0]. Overall, the prevalence of disposable e‐cigarette use increased from 0.08 to 1.85% (Table 1; PR = 22.3; 95% CI = 10.8–48.8).

**TABLE 1:** Age‐specific trends in current vaping, smoking and disposable e‐cigarette vaping prevalence in Great Britain.

	Prevalence		
	January 21	April 22	Prevalence ratio (95% CI)
Currently using inhaled nicotine (vaped or smoked)			
18‐year‐olds	30.2%	29.7%	0.99 (0.80–1.22)
25‐year‐olds	28.7%	30.3%	1.06 (0.94–1.19)
35‐year‐olds	25.6%	28.6%	1.12 (1.01–1.23)
45‐year‐olds	21.6%	24.1%	1.11 (0.99–1.24)
All adults	20.0%	21.2%	1.06 (0.92–1.22)
Currently vaping			
18‐year‐olds	11.3%	17.7%	1.57 (1.12–2.29)
25‐year‐olds	10.7%	15.2%	1.42 (1.16–1.77)
35‐year‐olds	9.4%	11.6%	1.23 (1.03–1.47)
45‐year‐olds	7.6%	8.1%	1.08 (0.88–1.33)
All adults	7.0%	8.2%	1.17 (1.01–1.35)
Currently smoking			
18‐year‐olds	24.5%	19.5%	0.80 (0.63–1.04)
25‐year‐olds	22.7%	19.9%	0.88 (0.76–1.02)
35‐year‐olds	19.7%	19.0%	0.97 (0.85–1.10)
45‐year‐olds	16.2%	16.3%	1.01 (0.88–1.16)
All adults	15.2%	14.5%	0.95 (0.87–1.05)
Currently vaping disposables			
18‐year‐olds	0.1%	10.7%	214 (56.7–5590)
25‐year‐olds	0.1%	4.7%	45.1 (17.1–247)
35‐year‐olds	0.2%	1.8%	9.84 (3.25–35.9)
45‐year‐olds	0.2%	0.9%	5.74 (2.57–22.2)
All adults	0.1%	1.9%	22.3 (10.8–48.8)

Weighted prevalence estimates from logistic regression allowing an interaction between age and month, modelled non‐linearly using restricted cubic splines (three knots). Data, analysis code and estimates for other months are available on‐line (https://osf.io/km3x6/).

**FIGURE 1:**
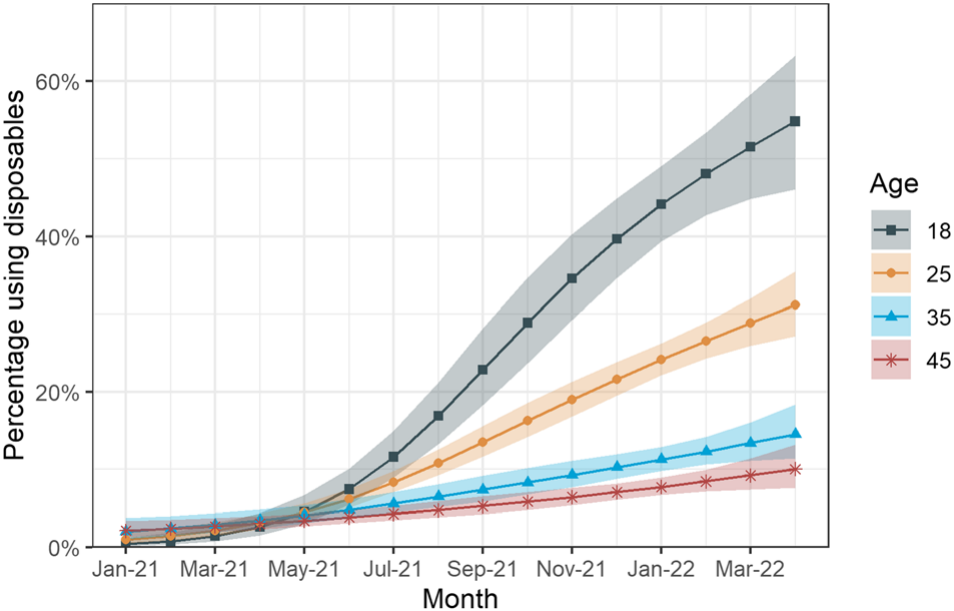
Percentage of current vapers using disposable e‐cigarettes across ages in Great Britain from 2021 to April 2022. A total of 36 876 eligible adults were surveyed (approximately 2300 each month). Lines represent point estimates from logistic regression allowing an interaction between age and month, modelled non‐linearly using restricted cubic splines (three knots). Shaded areas represent standard errors. Data and analysis code are available on‐line (https://osf.io/km3x6/)

Growth in disposable e‐cigarette vaping was most pronounced in the youngest participants (Fig. 1; interaction *P*‐value = 0.013). For instance, prevalence of disposable use among 45‐year‐old vapers rose from 2.1 to 10.0% (PR = 4.73; 95% CI = 2.06–23.6), whereas among 18‐year‐old vapers it increased from 0.4 to 54.8% (PR = 129; 95% CI = 28.5–4520).

Despite this, the overall percentage of adults currently using any inhaled nicotine (smoked or vaped) was relatively stable during the study period (Table 1; 20.0 versus 21.2%; PR = 1.06; 95% CI = 0.92–1.22). Among young adults, where the rise in disposable vaping was most pronounced, inhaled nicotine use changed little over time, estimated to be 30.2% for 18‐year‐olds in January 2021 and 29.7% April 2022 (Table 1; PR = 0.99; 95% CI = 0.80–1.22). However, during the period vaping prevalence rose from 11.3 to 17.7% among 18‐year‐olds (Table 1; PR = 1.57; 95% CI = 1.12–2.29); there was an uncertain decline in smoking prevalence from 24.5 to 19.5% (Table 1; PR = 0.80; 95% CI = 0.63–1.04). Conversely, in ages where vaping prevalence did not substantially increase, there appeared to be little change in smoking. For instance, the prevalence of vaping (Table 1; PR = 1.08; 95% CI = 0.88–1.33) and smoking (Table 1; PR = 1.01; 95% CI = 0.88–1.16) among 45‐year‐olds were relatively stable over time. More detailed monthly trends in the prevalence of inhaled nicotine use, vaping and smoking among adults of different ages are shown in Supporting information, Figs S1–S3.

Most disposable e‐cigarette vapers were current (71.6%) or former smokers (18.8%), with few reporting never having smoked regularly (9.6%). The proportion of disposable vapers who also smoked was similar across ages, but it may have declined slightly over time (Supporting information, Figs S4 and S5).

## DISCUSSION

Use of disposable e‐cigarettes rose sharply between 2021 and 2022 in Great Britain—with the most rapid growth observed among the youngest adults, mirroring trends observed in US adolescents [2]. At the start of 2021, fewer than 1% of 18‐year‐old vapers used disposables. This increased substantially throughout the past year, such that currently more than half of 18‐year‐old vapers report mainly using disposables. Despite this, the overall percentage of young people using any form of inhaled nicotine was stable over time, with an increase in vaping and an uncertain decline in smoking among young adults. This suggests that, in Great Britain up to 2022, disposable e‐cigarettes have primarily attracted those who would otherwise use rechargeable devices or cigarettes, rather than those who would otherwise not use any nicotine product. Nonetheless, patterns of nicotine product use can change rapidly. Early and routine publication of data such as these are needed to guide policy and research. Study limitations include the wide 95% CIs around PRs due to few participants reporting disposable e‐cigarette use in early months. The measure of disposable e‐cigarette vaping also did not distinguish between modern ‘bar’ style disposables from older ‘cigalikes’. Moreover, it asked about which type of e‐cigarette vapers ‘mainly’ use, so vapers who used disposables as a secondary product were not captured; therefore, the estimated prevalence of disposable vaping actually represents a lower bound for the true prevalence. Future studies should examine why disposable e‐cigarettes have become the product of choice among young people in Great Britain and the United States [2] and whether similar trends have occurred in other countries.

## DECLARATION OF INTERESTS

H.T.B., L.K., M.D. and S.J. declare no conflicts of interest. J.B. has received unrestricted research funding to study smoking cessation from manufacturers of smoking cessation medications (Pfizer and Johnson & Johnson).

## AUTHOR CONTRIBUTIONS

**Harry Tattan‐Birch:** Conceptualization‐Lead; formal analysis‐lead; investigation‐lead; methodology‐lead; writing – original draft‐lead; writing – review & editing‐lead. **Sarah Jackson:** Conceptualization; investigation; methodology; supervision. **Loren Kock:** Conceptualization; data curation; investigation; methodology. **Martin Dockrell:** Conceptualization; investigation; supervision. **Jamie Brown:** Conceptualization; data curation; investigation; methodology; supervision.

## Supporting information


**Figure S1.** Smoking prevalence across ages in Great Britain from 2021 to April 2022.
**Figure S2.** Vaping prevalence across ages in Great Britain from 2021 to April 2022.
**Figure S3.** Prevalence of inhaled nicotine use (smoking/vaping) across ages in Great Britain from 2021 to April 2022.
**Figure S4.** Percentage of disposable vapers who currently smoke across ages in Great Britain.
**Figure S5.** Percentage of disposable vapers who currently smoke across months from 2021 to April 2022 in Great Britain.
**Figure S6.** Percentage of non‐disposable vapers who currently smoke across ages in Great Britain.
