# Supplementary material for: Rapid growth in disposable e‐cigarette vaping among young adults in Great Britain from 2021 to 2022: a repeat cross‐sectional survey
Source: Addiction. 2022 Sep 11;118(2):382–6. doi: 10.1111/add.16044 (PMC10086805; doi:10.1111/add.16044)
Supplement: Supplementary file 1 — Figure S1. Smoking prevalence across ages in Great Britain from 2021 to April 2022. Figure S2. Vaping prevalence across ages in Great Britain from 2021 to April 2022. Figure S3. Prevalence of inhaled nicotine use (smoking/vaping) across ages in Great Britain from 2021 to April 2022. Figure S4. Percentage of disposable vapers who currently smoke across ages in Great Britain. Figure S5. Percentage of disposable vapers who currently smoke across months from 2021 to April 2022 in Great Britain. Figure S6. Percentage of non‐disposable vapers who currently smoke across ages in Great Britain. [file ADD-118-382-s001.docx]

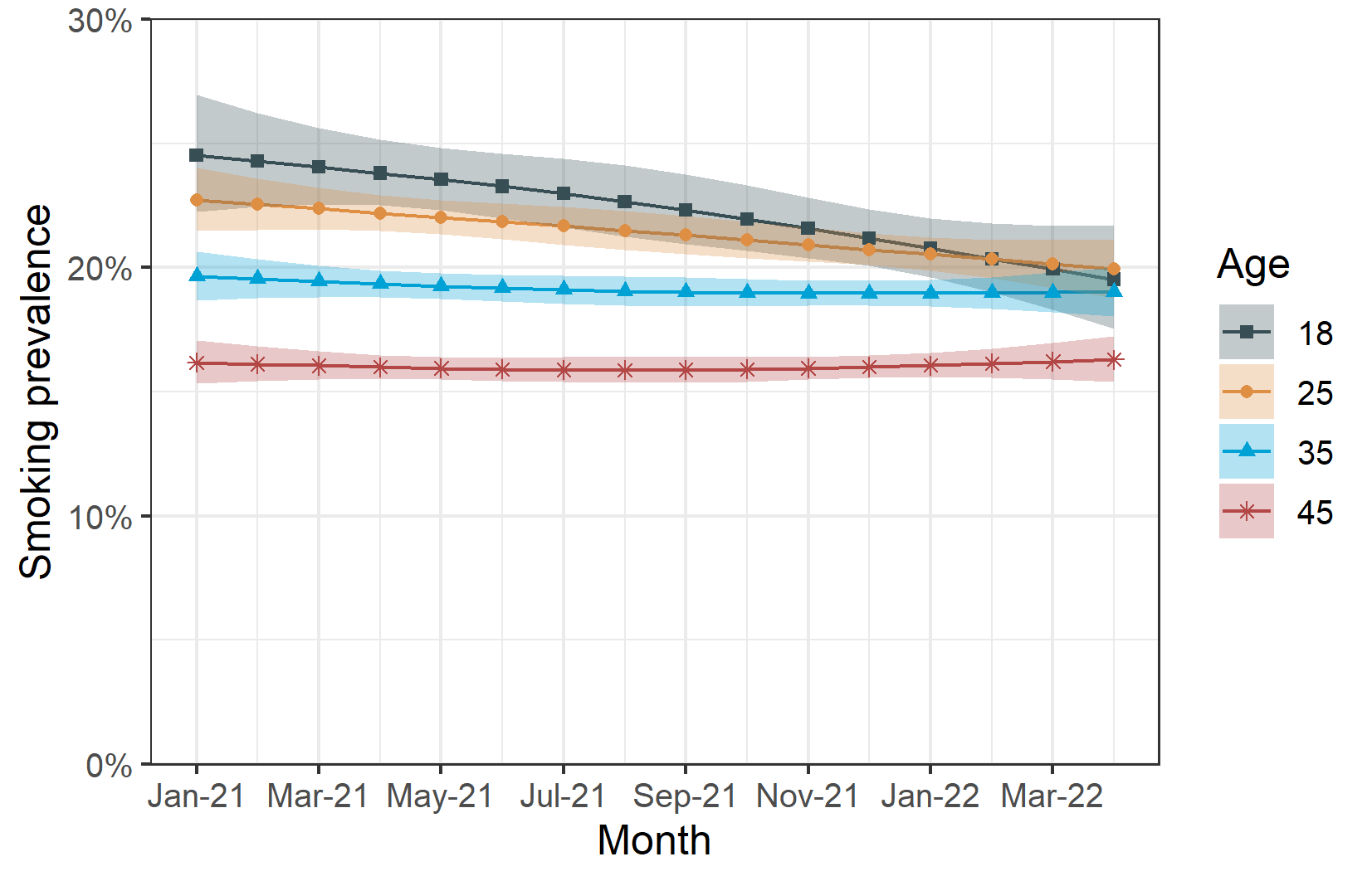


**Supplementary Figure 1. Smoking prevalence across ages in Great Britain from 2021 to April 2022.** A total of 36,876 eligible adults were surveyed (approximately 2,300 each month). Lines represent point estimates from logistic regression allowing an interaction between age and month, modelled non-linearly using restricted cubic splines. Shaded areas represent standard errors.


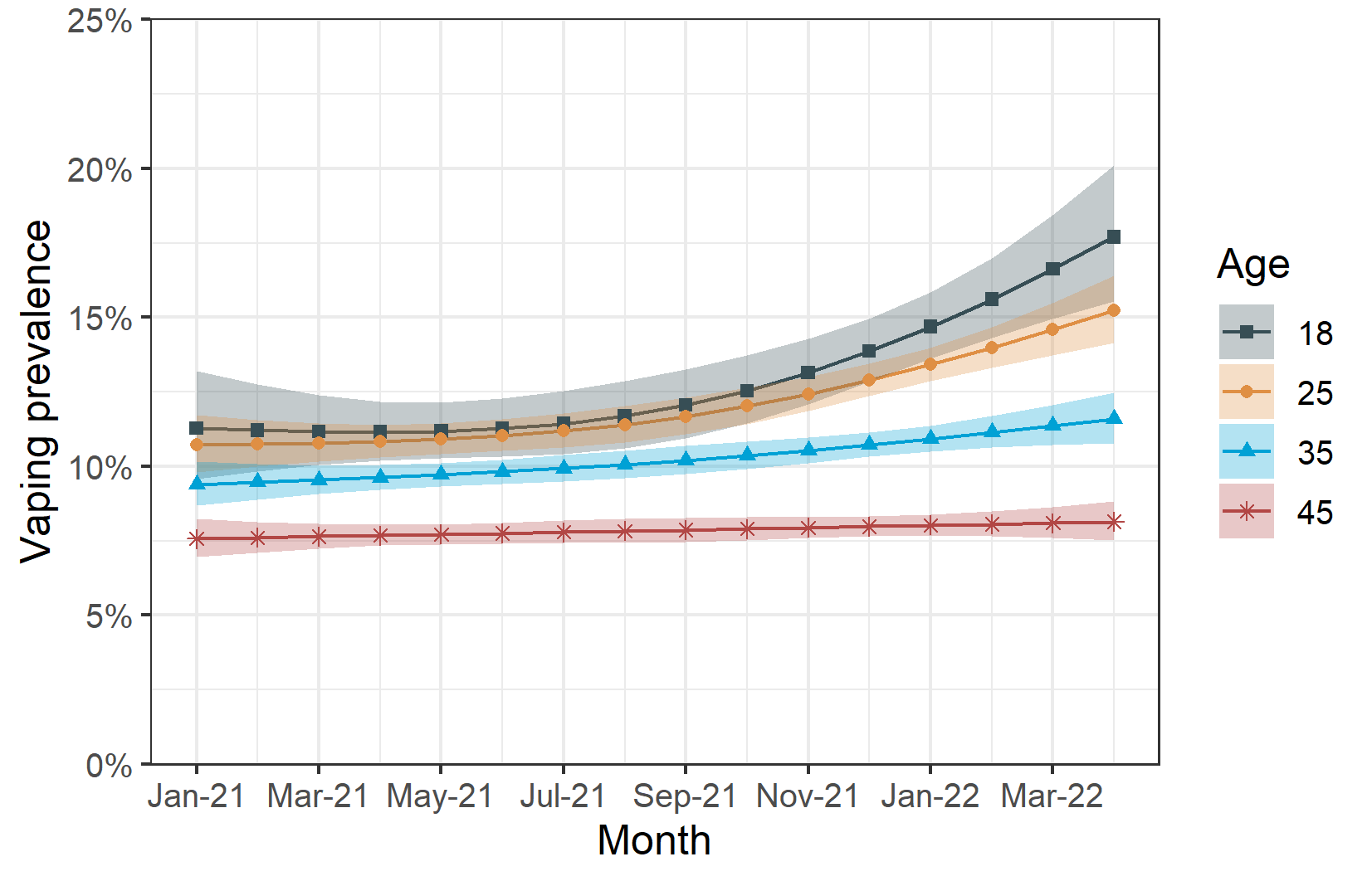


**Supplementary Figure 2. Vaping prevalence across ages in Great Britain from 2021 to April 2022.** A total of 36,876 eligible adults were surveyed (approximately 2300 each month). Lines represent point estimates from logistic regression allowing an interaction between age and month, modelled non-linearly using restricted cubic splines. Shaded areas represent standard errors.


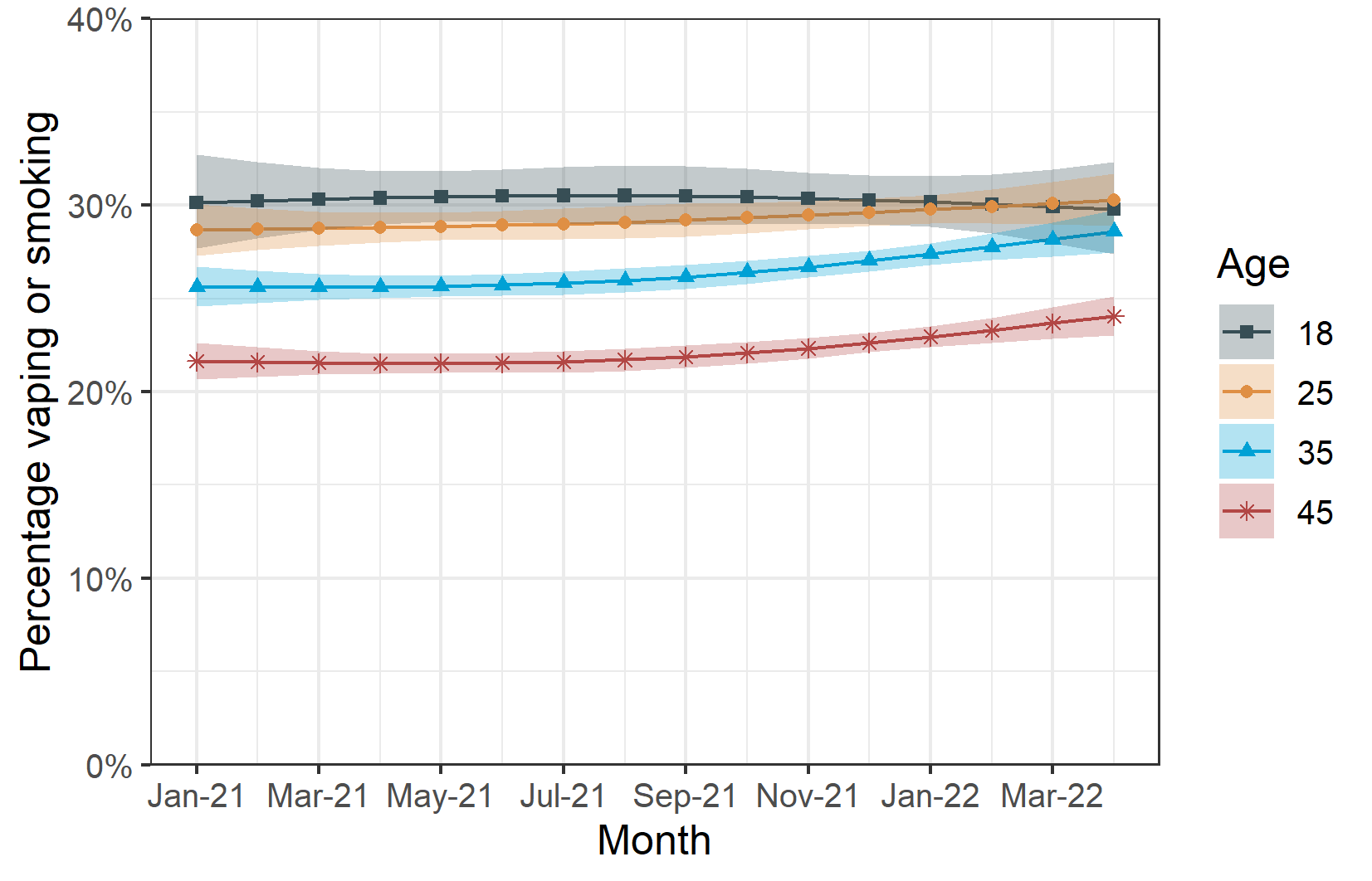


**Supplementary Figure 3. Prevalence of inhaled nicotine use (smoking/vaping) across ages in Great Britain from 2021 to April 2022.** A total of 36,876 adults were surveyed (approximately 2,300 each month). Lines represent point estimates from logistic regression allowing an interaction between age and month, modelled non-linearly using restricted cubic splines. Shaded areas represent standard errors.


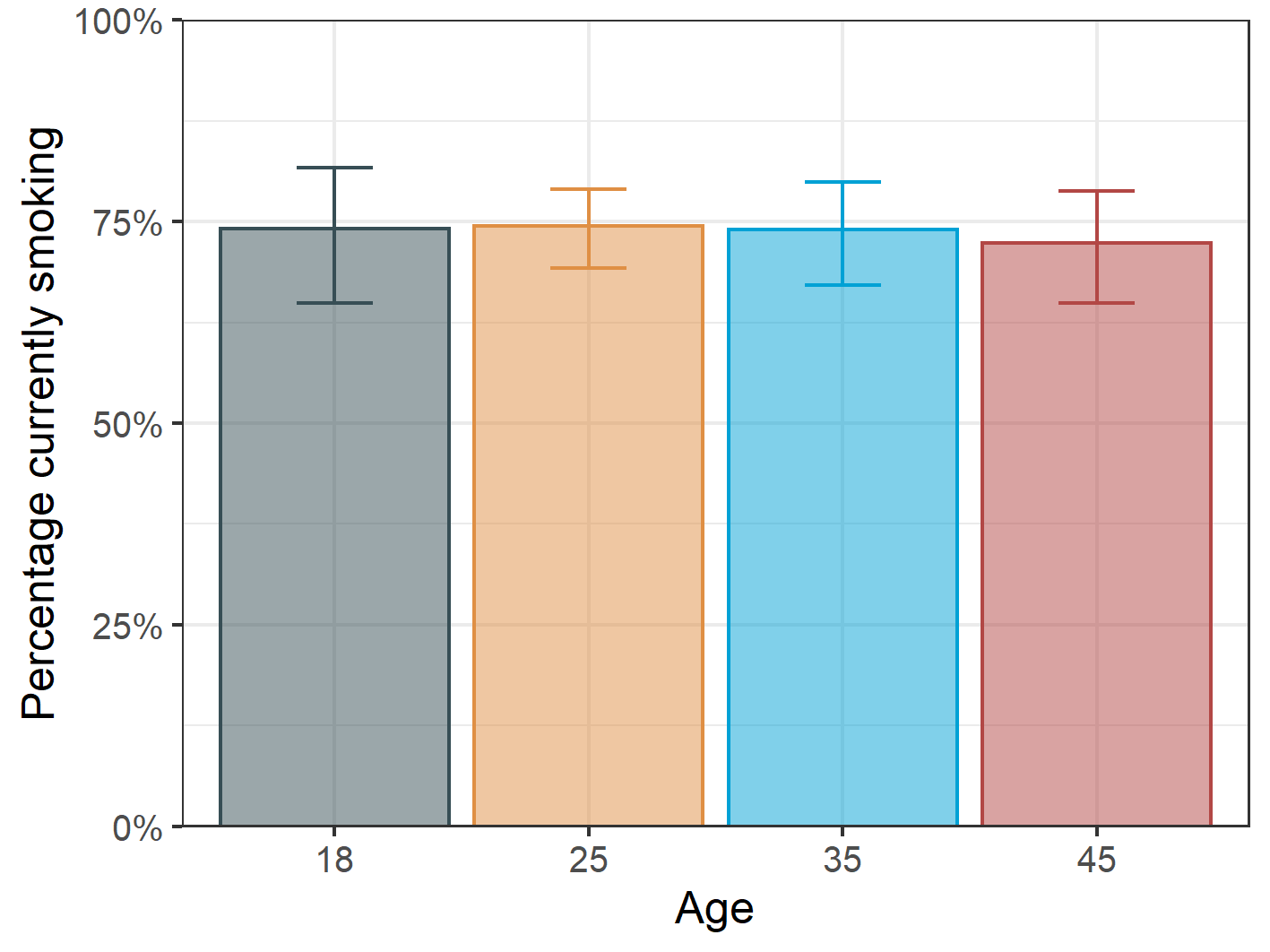


**Supplementary Figure 4. Percentage of disposable vapers who currently smoke across ages in Great Britain.** Height of bars represent point estimates from logistic regression with age modelled non-linearly using restricted cubic splines. Error bars represent standard errors.


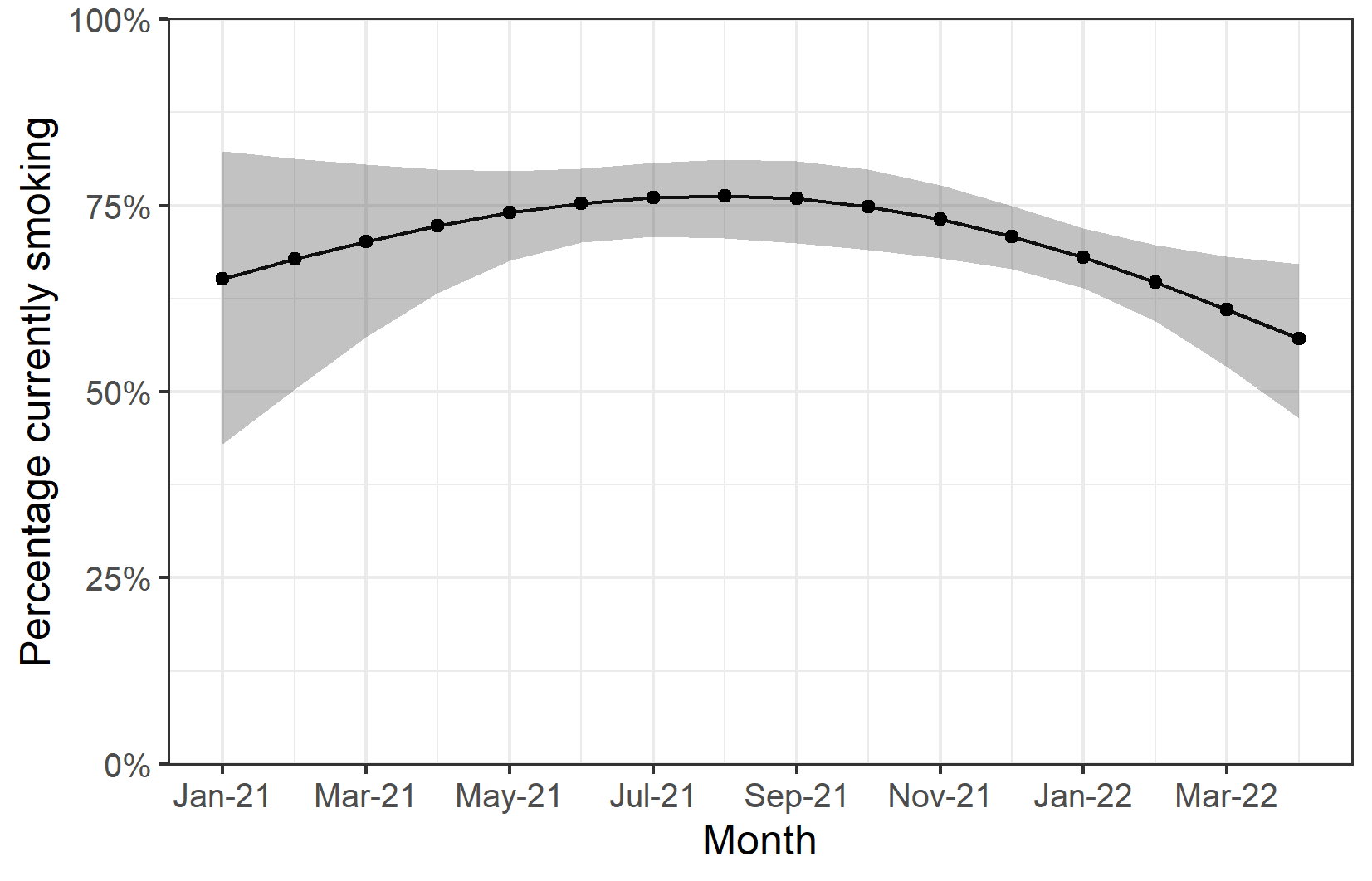


**Supplementary Figure 5. Percentage of disposable vapers who currently smoke across months from 2021 to April 2022 in Great Britain.** Line represents point estimates from logistic regression with month modelled non-linearly using restricted cubic splines. Shaded bands represent standard errors.


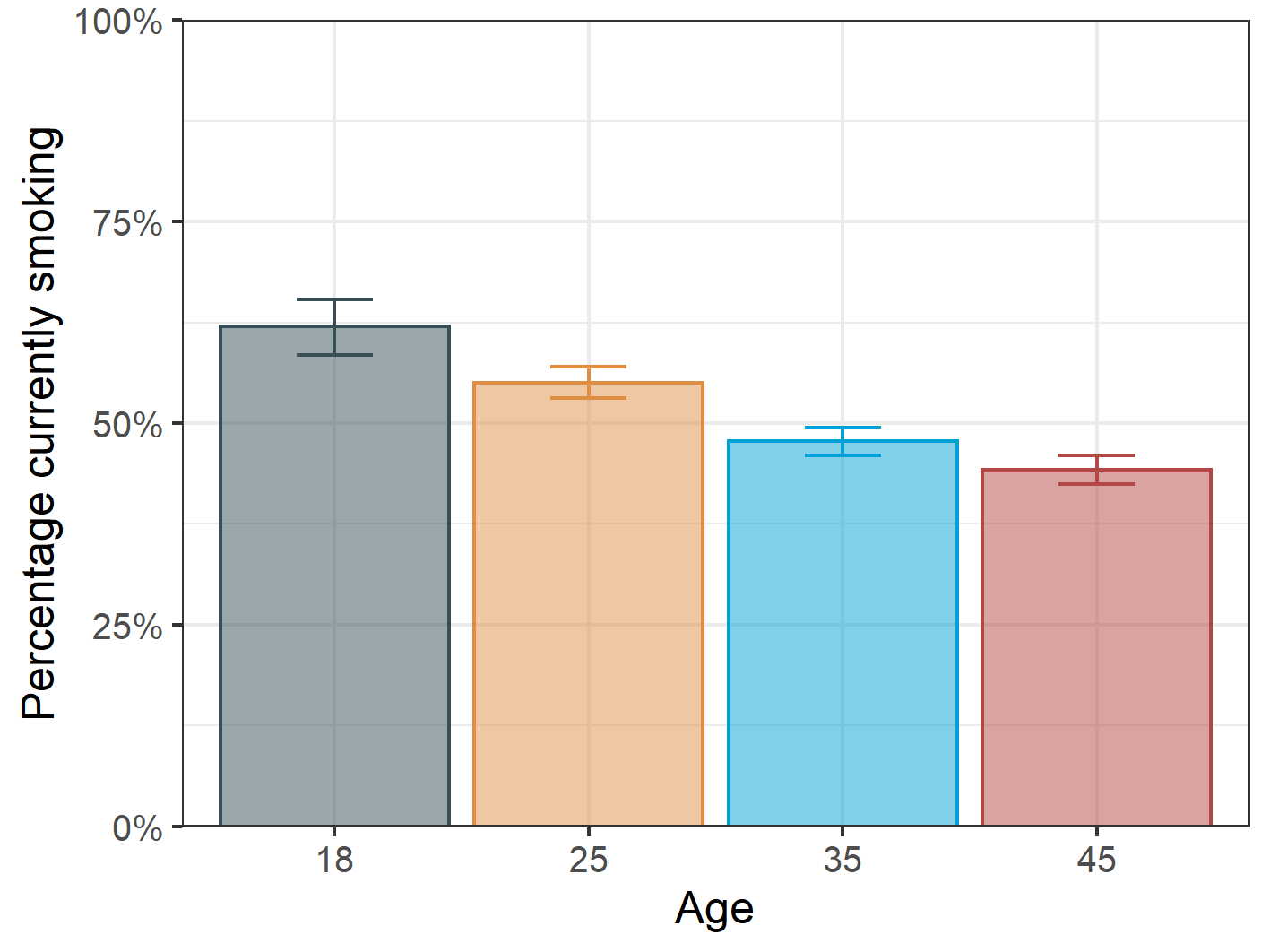


**Supplementary Figure 6. Percentage of non-disposable vapers who currently smoke across ages in Great Britain.** Height of bars represent point estimates from logistic regression with age modelled non-linearly using restricted cubic splines. Error bars represent standard errors.
